# A Reinforcement Learning Routing Protocol for UAV Aided Public Safety Networks

**DOI:** 10.3390/s21124121

**Published:** 2021-06-15

**Authors:** Hassan Ishtiaq Minhas, Rizwan Ahmad, Waqas Ahmed, Maham Waheed, Muhammad Mahtab Alam, Sufi Tabassum Gul

**Affiliations:** 1 School of Electrical Engineering and Computer Science, National University of Sciences and Technology (NUST), Islamabad 44000, Pakistan; hminhas.msee17seecs@seecs.edu.pk (H.I.M.); maham.waheed@seecs.edu.pk (M.W.); 2 Department of Electrical Engineering, Pakistan Institute of Engineering and Applied Sciences (PIEAS), Islamabad 45650, Pakistan; waqas@pieas.edu.pk (W.A.); tabassumgul@gmail.com (S.T.G.); 3 Thomas Johann Seebeck Department of Electronics, Tallinn University of Technology, 19086 Tallinn, Estonia; muhammad.alam@taltech.ee

**Keywords:** clustering, d2d communication, disasters, energy conservation, network lifetime, public safety networks, reinforcement learning

## Abstract

In Public Safety Networks (PSNs), the conservation of on-scene device energy is critical to ensure long term connectivity to first responders. Due to the limited transmit power, this connectivity can be ensured by enabling continuous cooperation among on-scene devices through multipath routing. In this paper, we present a Reinforcement Learning (RL) and Unmanned Aerial Vehicle- (UAV) aided multipath routing scheme for PSNs. The aim is to increase network lifetime by improving the Energy Efficiency (EE) of the PSN. First, network configurations are generated by using different clustering schemes. The RL is then applied to configure the routing topology that considers both the immediate energy cost and the total distance cost of the transmission path. The performance of these schemes are analyzed in terms of throughput, energy consumption, number of dead nodes, delay, packet delivery ratio, number of cluster head changes, number of control packets, and EE. The results showed an improvement of approximately 42% in EE of the clustering scheme when compared with non-clustering schemes. Furthermore, the impact of UAV trajectory and the number of UAVs are jointly analyzed by considering various trajectory scenarios around the disaster area. The EE can be further improved by 27% using Two UAVs on Opposite Axis of the building and moving in the Opposite directions (TUOAO) when compared to a single UAV scheme. The result showed that although the number of control packets in both the single and two UAV scenarios are comparable, the total number of CH changes are significantly different.

## 1. Introduction

Man-made disasters such as terrorism can result in both the loss of life and critical infrastructure. It is estimated that an underdeveloped country like Pakistan incurred direct losses of 127 billion dollars in the last 17 years or so due to terrorism [1]. In addition, the attacks like Army Public School (APS) Peshawar, in which 150 students lost their lives, left a huge social and psychological impact on society [2]. This event has resulted in the complete overhaul of the security infrastructure and caused indirect losses to the economy. Numerous other terrorist incidents such as on the Pakistan Navy Ship (PNS) Mehran, and General Headquarter (GHQ) are examples in which terrorists attacked a building, took hostages, and/or destroyed critical infrastructure. In these scenarios, to disrupt coordination, communication/cellular infrastructure is often taken out either by the authorities or terrorists. The on-scene devices carried by the trapped victims are unable to communicate to first responders or law enforcement agencies. The information (location and number of victims, audio, video, and images) captured by on-scene available devices can provide timely information to first responders for carrying out a coordinated rescue operation [3,4].

**Figure 1:**
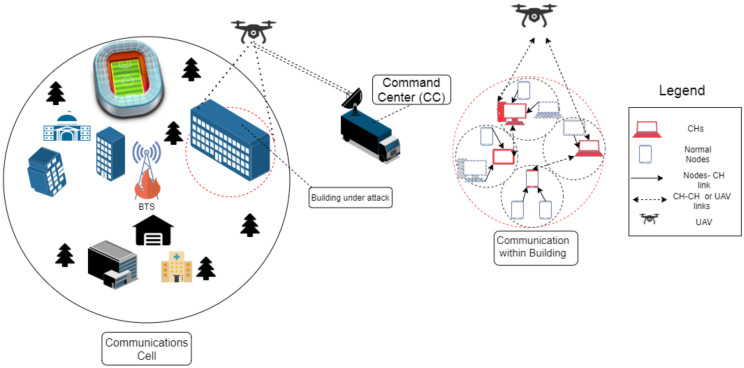
Disaster-affected region.

In these situations, a Device to Device (D2D) network provides an alternative method of communication and connectivity among devices [5,6]. The D2D network along with the presence of an Unmanned Aerial Vehicle (UAV) can ensure the information collected by the devices to reach the Command Center (CC). This situation is depicted in Figure 1. Since the transmission power of the devices are limited, it may be possible that some of the devices are unable to reach the UAV. It therefore becomes imperative to cluster the devices. Clustering is a process in which the network is divided into small substructures called clusters, based on node degree, mobility, weights, etc., [7]. This small substructure consists of Cluster Members (CMs) and a Cluster Head (CH). In the network, the CMs communicate with their respective CHs to forward their data to the UAV. The CHs can also rely on each other in forwarding data to the UAV and subsequently to the CC.

Multiple clustering techniques are studied in the literature based on different applications [8,9,10,11,12,13,14,15,16,17,18,19,20]. For example, the Link Cluster Algorithm (LCA) introduces the concept of a Gateway (GW) node to provide better connectivity among neighboring CHs [8]. However, this scheme is unstable due to numerous ID exchanges between nodes. The Least Cluster Change (LCC) [9] reduces the cost of re-clustering, which gives stability to the clusters. In [10], the authors proposed a new underlay clustering-based D2D network for the partial and disconnected network. It forms dynamic clusters using adhoc base stations or mobile devices. In [11], low frame-sized beacons signals are broadcasted by CHs to decrease signaling overhead. CMs are declared based on the SINR values. The α-Stability Structure Clustering algorithm (α-SSCA) is proposed in [12] in which CH is selected based on a score function calculated by the exchange of hello messages between neighboring nodes. The concept of quasi clusters, a special cluster within a cluster, are introduced in [13] to help in reducing transmission power resulting in a longer network lifetime.

In [15], the authors proposed a new clustering scheme in which first clusters are formed by dividing the area into multiple small partitions and then selecting the CH in those partitions. These will be selected based on energy. To prolong network lifetime and to avoid a blind spot issue in the scheme, re-clustering is performed when the energy of the existing CH reaches a certain threshold. This scheme suffer from scalability issues. In our earlier work [16], we compared different basic clustering schemes such as Clustering without GW (CG), Clustering with GW (CWG), and No Clustering (NC) in terms of throughput and energy. Simulation results show that the performance of CWG is best in terms of throughput and energy.

Another important clustering technique is called the K mean clustering, which is mostly used in Wireless Sensor Networks (WSNs). In [17], K means and LEACH-C are combined to prolong the lifetime of the network. At the start, the K mean algorithm is used to form clusters, then LEACH-C is applied to each cluster. The method reduces the overhead and increases the packet success rate. In [18], the authors have used the K means L layer algorithm, which results in a decreased number of clusters enhancing network lifetime. In [19], an energy-efficient K mean clustering protocol is proposed to optimize packet size based on the channel conditions. This approach reduces energy consumption and increases the overall network lifetime. In [20], an optimum value of K is obtained using the elbow method and afterwards, clustering will be applied using the K mean algorithm. Simulations show that after running the elbow method on different points, there comes a point where gain gained by increasing K will drop. This is the optimum value of K.

In the absence of a cellular network, relaying critical data from the devices to the CC is another important concern. Several routing protocols/algorithms exist in the literature for multi-hop/multi-user networks. An emergency routing technique based on body-to-body networks, known as an Optimized Routing Approach for Critical and Emergency Networks (ORACE-NET) is proposed in [21]. It establishes temporary network connectivity for relief works in disaster-affected areas. The results show that ORACE-NET performs better in terms of energy consumption and throughput. Considering network size, different routing schemes perform differently. Interference Aware Routing (IAR), Shortest Path Routing (SPR), and Broadcast Routing (BR) are tested in [22] to find the shortest emergency route in a disaster scenario. The simulation results show that the BR has the highest packet success ratio for small networks, while IAR performs better for large networks. In [23], the authors applied Simultaneous Wireless Information and Power Transfer (SWIPT) on CHs to achieve better performance in a disaster-affected region.

Once the devices are clustered, the UAV position and trajectory plays a crucial role in determining the Energy Efficiency (EE) of the network. Mainly, in the disaster situation the UAV acts as a relay node for on-scene devices [24,25,26,27,28,29,30,31,32,33]. For UAV deployment in PSN, the authors in [27] discovered the optimal altitude for a UAV that maximizes coverage. In [29], authors proposed a UAV-assisted solution to establish energy-efficient connectivity in a disaster-affected region in the presence of Critical Nodes (CNs). In [30], authors proposed a D2D-based solution using UAVs that can reduce the response time significantly. In [31], authors used Reinforcement Learning (RL) technique to deploy UAVs in a disaster scenario to maximize total user coverage. In [32], the intelligent placement of UAVs as temporary aerial base stations is discussed for public safety communications. In [34], authors proposed a UAV-assisted vehicular communication framework using Software Defined Networking (SDN) to reduce the processing cost of vehicles. UAV will act as a flying relay and helps in forwarding data to a Mobile Edge Computing (MEC) server. This algorithm reduces the average system cost by half. In [35], the authors proposed a new cellular network for UAVs to support a high data rate. Three transmission modes of UAV with a network, UAVs, and devices i.e., U2N, U2U, and U2D are studied. Authors in [36] proposed a multi agent deep RL-based UAV framework assisted by MEC in which UAVs will assist users on the ground. Results showed considerable gain in terms of fairness and energy consumption. In this paper [37], the authors used UAVs in a disaster environment to extract information from its one-hop devices using wireless power transfer technique. The graph traversal method is used to reduce the energy cost of the UAV to one third of the total energy.

In [38], authors reviewed and discussed different UAV-Aided Wireless Sensor Networks (UAWSNs). The advantage of these networks is increased coverage and maximum energy consumption at the cost of variable paths and mobility issues resulting in coverage problems in these networks. In [39,40,41,42,43,44,45,46], different UAV WSN-structured routing (flat, cluster-based, tree-based, and location-based) protocols are proposed. Authors in [47] used UAV communication to provide rescue operations in disaster-affected areas. UAVs are spread over the entire area to provide network coverage. Gateway UAVs are further used to deliver the information to the main network. In this work, the aim is to maximize the data rate while considering battery consumption. To address the problem of gateway UAV selection, authors in [48] proposed a gateway UAV selection algorithm named Battery-Aware Multi Arm Bandit (BA-MAB). They have also explored the use of machine learning. Two kinds of UAVs are present in this work: Access UAVs and gateway UAVs. The objective is to maximize the data rate while consuming minimum energy.

In [49], authors used drones for surveillance and data collection in buildings. Sensors are used by the drones to navigate the buildings to identify and pinpoint the problems. Deep RL is applied along with curriculum learning and neural networks.

Existing work on UAV and clustering mainly focuses on optimizing data collection in WSNs. However, as demonstrated from the above literature, PSN is another important use of UAVs and clustering simultaneously. The energy dynamics of on-scene devices in a disaster scenario is highly dynamic compared to other uses, which amplifies the complexity of ensuring end-to-end connectivity. Therefore, in this paper:

- We first analyze the impact of different clustering schemes and a UAV presence on the performance of multihop routing in a disaster scenario. We then present a RL approach to ensure end-to-end connectivity and improve Energy Efficeincy (EE) of PSNs.
- We consider the mobility of UAVs around the disaster area. Multiple UAV trajectories are devised in order to improve the coverage of clusters in the disaster area while ensuring EE.

This paper is organized as follows: In Section 2, the detailed system model is presented. Section 2.1 presents network throughput and delay, Section 2.2 presents the energy model, and the problem formulation is presented in Section 2.3. In Section 3, routing methods are discussed in detail with clustering, route discovery, routing, and control overhead in Section 3.1, Section 3.2, Section 3.3 and Section 3.4 respectively. Section 4 provides a comparison of clustering schemes with respect to energy and throughput. Section 4.1 and Section 4.2 discuss reinforcement-based routing and combined RL and UAVs trajectory optimization. In the end, Section 5 gives the conclusion and future directions.

## 2. System Model

**Figure 2:**
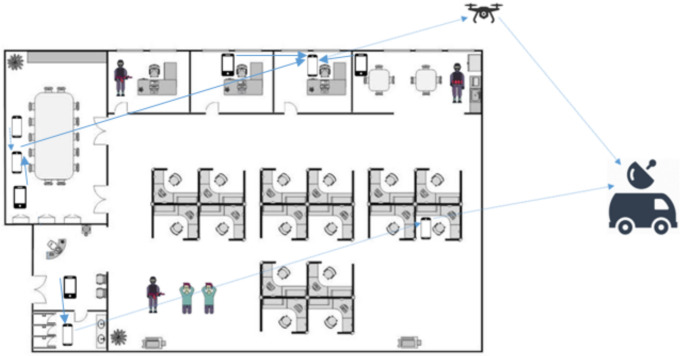
Disaster hit area with Unmanned Aerial Vehicle (UAV) and Command Center (CC).

We consider a man-made disaster scenario in which terrorists attack a large building. It is assumed that the normal cellular/wireless infrastructure is either blocked by security forces or destroyed by the terrorists. We further assume the presence of some on-scene devices (from here on called nodes) held by the trapped victims. If provided with an adequate emergency communication network, the information carried by these nodes can yield great insight for the law enforcement agencies. However, it may not be possible for the emergency communication network to provide coverage to all the nodes simultaneously. In this situation, the nodes can cooperatively communicate with each other to form a D2D multi-hop network. To simplify communication between multiple nodes, the devices can form clusters assisted through the D2D network. The UAV and CC are deployed outside the building perimeter to collect the information from these clusters. The clusters within range of CC communicate directly with the CC and the clusters outside the range of the CC communicate with the CC through a UAV. This situation is depicted in Figure 2, in which the UAV (acting as a relay) is placed outside the building to connect the nodes with the CC used by law enforcement agencies. To protect the CC from an ambush by the terrorists, the CC is deployed slightly away from the building. The symbols are mentioned in Table 1.

**Figure 3:**
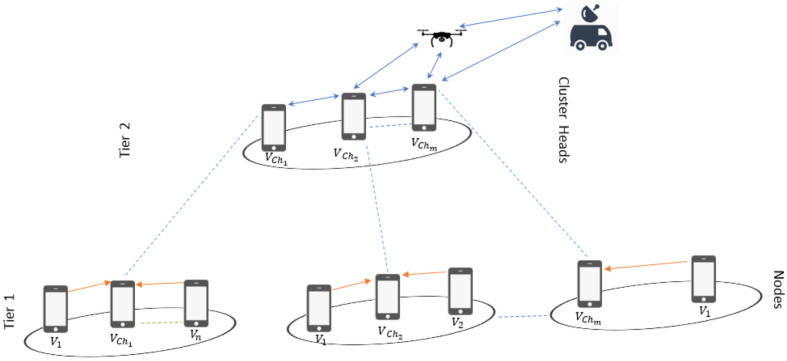
Two-tier graph.

Figure 3 shows a two-tier network with *N* nodes that are randomly distributed in 100 m × 100 m. The nodes are divided into *i* clusters and the set of nodes in each cluster is denoted by $N_{i}$, where i={1,…,I}. Considering a limitation on the transmit power of on-scene devices, the maximum distance between CH and its CM is restricted to $R_{max}$. Whereas, a CH in this network can communicate with the other CHs over a maximum distance denoted by $T_{max}$. The pathloss [50] between links separated by distance *d* is calculated from:

(1) $$PL\left(d\right)=20\times log_{10}\left(d\right)+46.4+20\times log_{10}\left(f_{c}/5\right)+12\times n_{w}+17+4\left(n_{f}-1\right)$$

where the path loss exponent is assumed to be 2, $f_{c}$ is the carrier frequency, $n_{w}$ is the number of walls (taken as 1), and $n_{f}$ is the number of floors (taken as 0) as we assume that the devices are on the same floor.

### 2.1. Network Throughput and Delay

In this scenario, the total network throughput (*r*) is the sum of throughput from each CM to the CC. Given $d_{mn}$ represents the link distance between *m* and *n*, then the throughput is defined as:

(2) $$\begin{array}{c} r_{mn}=B\log_{2}\left(1+\frac{P_{t}}{N_{o}PL\left(d_{mn}\right)}\right). \end{array}$$

The CHs in the network can cooperate with each other to forward their data to the UAV or CC. Since a CM in cluster *i* ($s_{i}\in N_{i}$) takes multiple hops to reach CC, we define a routing matrix for each CM, denoted by $H_{i,s_{i}}$. It is assumed that the routing matrix remains the same for some time horizon and also remains the same for all the nodes in a cluster. Therefore, the routing matrix is defined as $H_{i,s_{i}}=\left(h_{mn}\right)\in R^{\left(K+1)\times (K+2\right)}$, where $h_{mn}$ denotes the status of the connection between *m* and *n*, $m\in \{s_{i},{\mathrm{CH}}_{1},\ldots ,{\mathrm{CH}}_{I},\mathrm{UAV},\mathrm{CC}\}$ and $n\in \{{\mathrm{CH}}_{1},\ldots ,{\mathrm{CH}}_{I},\mathrm{UAV},\mathrm{CC}\}$, and $h_{mn}=1$ ($h_{mn}=0$) indicates the presence (absence) of path between the nodes *m* and *n*. For example, if the 2nd node in the 1st cluster forward their data to CC through CH${}_{3}$ and UAV, then the routing matrix for such a configuration can be written as:

(3) $$\begin{array}{c} H_{1,2}=\left[\begin{array}{ccccc} 1 & 0 & 0 & 0 & 0\\ 0 & 0 & 1 & 0 & 0\\ 0 & 0 & 0 & 1 & 0\\ 0 & 0 & 0 & 0 & 1 \end{array}\right]. \end{array}$$

The first entry $h_{11}=1$ indicates the link between the 2nd node and 1st CH, $h_{23}=1$ indicates the link condition between CH${}_{1}$ and CH${}_{3}$, $h_{34}=1$ connects CH${}_{3}$ to the UAV, and $h_{45}=1$ successfully terminates the information at CC.

Let ${\hat{\zeta }}_{i,s_{i}}$ defines the throughput for all the possible paths from the node in $s_{i}$, then the throughput on the paths defined by the routing matrix $H_{i,s_{i}}$ can be written as ${\hat{\zeta }}_{i,s_{i}}=\left(r_{mn}h_{mn}\right)\in R^{\left(K+1)\times (K+2\right)}$. Since a node takes multiple hops to reach CC, we assume that the minimum throughput in all the multi-hop links is the throughput of the node. Let $\lambda_{i,s_{i}}$ be the minimum value of ${\hat{\zeta }}_{i,s_{i}}$, then the sum throughput of all the nodes can be written as:

(4) $$\begin{array}{c} \zeta =\sum_{i\in I}\sum_{s_{i}}\lambda_{i,s_{i}}. \end{array}$$

The number of hops can be directly computed from the rank of $H_{i,s_{i}}$ denoted by ${℘}_{i,s_{i}}$. Using $\lambda_{i,s_{i}}$, packet size *L*, and transmission rate, the end-to-end delay (ignoring propagation delay) of a node indexed by $s_{i}$ in cluster *i* can be calculated as:

(5) $$\begin{array}{c} \delta_{i,s_{i}}=\sum_{m}\sum_{n}\frac{Lh_{mn}}{r_{mn}}, \end{array}$$

whereas, the sum end-to-end delay of all nodes in the network are given as:

(6) $$\begin{array}{c} \Delta =\sum_{i\in I}\sum_{s_{i}}\delta_{i,s_{i}}. \end{array}$$

### 2.2. Energy Model

Assuming that all the nodes transmit with constant power $P_{t}$, the transmission energy $E_{tx}\left(i,s_{i}\right)$ of CM (indexed by $s_{i}$ of cluster *i*) can be written as:

(7) $$\begin{array}{c} E_{tx}\left(i,s_{i}\right)=\frac{LP_{t}}{r_{s_{i}i}}. \end{array}$$

Assuming, a CM has an initial energy $E_{initial}\left(i,s_{i}\right)$, then the residual energy of the node after *T* transmissions becomes:

(8) $$\begin{array}{c} E_{res}\left(i,s_{i}\right)=E_{initial}\left(i,s_{i}\right)-\sum_{T}E_{tx}\left(i,s_{i}\right). \end{array}$$

Since a CH forwards data of all the CMs, the transmission energy of *i*th CH becomes:

(9) $$\begin{array}{c} E_{res(i)}=E_{initial}\left(i\right)-\sum_{T}\sum_{s_{i}}E_{tx}\left(i\right), \end{array}$$

where, $E_{initial}\left(i\right)$ is the initial energy of CH, and

(10) $$\begin{array}{c} E_{tx}\left(i\right)=\sum_{n}\frac{LP_{t}h_{in}}{r_{in}}. \end{array}$$

The total energy consumption in the networks after *T* transmissions can now be written as:

(11) $$\begin{array}{c} E_{tot}=\sum_{i\in I}\sum_{s_{i}}E_{tx}\left(i,s_{i}\right)+\sum_{i\in I}E_{tx}\left(i\right). \end{array}$$

### 2.3. Problem Formulation

The objective in this work is to maximize throughput, however, maximizing throughput can lead to higher energy consumption. Since the devices have limited energy, the higher energy consumption can lead to dead nodes in the network and subsequently network singularities. Therefore in this paper, we aim to increase EE. Based on the sum throughput and energy calculations (4) and (11) in the previous subsections, we can define the EE as:

(12) $$\begin{array}{c} \mathrm{EE}=\frac{\zeta }{E_{tot}}=\frac{\sum_{i\in I}\sum_{s_{i}}\lambda_{i,s_{i}}}{\sum_{i\in I}\sum_{s_{i}}E_{tx}\left(i,s_{i}\right)+\sum_{i\in I}E_{tx}\left(i\right)}. \end{array}$$

The objective function can now be written as:

(13) $$\begin{array}{cc} \max_{H_{i,s_{i}}}\; & \mathrm{EE}\\ s.t.\; & E_{res}\left(i\right)>0\\ \; & R_{max}\leq 30m. \end{array}$$

In the following we present an intelligent routing technique based on RL to maximize the EE.

## 3. Reinforcement Learning-Based Routing

To achieve the above objective function, we define a routing methodology which involves three steps. In the first step, the devices form clusters to decrease the transmission energy. In the second step, route discovery takes place and end-to-end paths are found. Note that routing discovery is dependent on the type of underlying clustering schemes as different clustering schemes generate different network configurations or end-to-end paths. In the last step, based on routing discovery we apply RL to determine transmission paths to improve the EE of the network.

As discussed earlier, clustering can impact route discovery, therefore, to optimize the route discovery we compare the performance of different clustering schemes.

### 3.1. Clustering

In this paper, we consider two types of clustering schemes: (1) Clustering-energy which is a distributed clustering scheme and (2) clustering-K mean which is a centralized clustering scheme.

#### 3.1.1. Clustering-Energy

In this scheme, we assume that the nodes have no prior information about the energy and distance of other nodes. Initially, a node will check its $E_{res}$, and based on its level decides about its current status as either CH or CM. If the node energy is 100 times above the energy threshold, then it will immediately declare itself as a CH. Otherwise, it will wait for a small interval equivalent to a single transmission round (for other nodes to declare themselves as CH) and then declares itself as a CH. If the node declares itself as a CH then it will broadcast its CH Identification (ID) and $E_{res}$ information. This CH ID will be selected randomly between 0 and $I_{max}$. This broadcast is limited by the $Tx_{range}=30$ m. The nodes which receive this broadcast will compare their own $E_{res}$ with the received information. If the $E_{res}$ of the node is higher compared to that of the current CH, it will declare itself as a CH and broadcast its own $E_{res}$ and CH ID. On receiving this broadcast, the node that has previously declared itself as a CH will change its status to CM and send an association request to the new CH. All the other nodes except CH which receive this association request will change their CH. On the other hand, if the $E_{res}$ of the receiving node is lower than the CH, then they will send an association request to the CH and change their status as member nodes.

Afterwards, the CH will broadcast its cluster ID at $Range=1.5\times Tx_{range}$. All the CHs that receive this broadcast will forward it until all the CHs receive this ID. If some CH have selected the same ID before, then this will resolve the issue because this broadcast and each CH will now have a unique CH-ID. The process of re-clustering will start when the $E_{res}$ of the CH reaches the energy threshold value. At that point the CH will broadcast a CH dead message. Nodes that receive this message will start the CH selection process.

We observe the nodes falling in the overlapping zones of two clusters can act as a GW node. This will provide an added degree of freedom in route discovery. Therefore, based on the above procedure, we derive another scheme called clustering-energy-GW. The only difference is that the GW nodes are formed if a member node receives the broadcast of two or more CHs. The node will set and broadcast its status as a GW node. These schemes are easy to implement and need no central authority, however, this is achieved at a higher cost of cluster formation and CH selection.

#### 3.1.2. Clustering-Kmean

K mean clustering is an unsupervised machine learning algorithm to cluster nodes in the network. From a network perspective, it is a centralized scheme and requires distance information of all the nodes. This algorithm comprises of two key steps. In the first step, K centres are placed randomly in the given geographic area and all nodes must associate themselves with the closest centre. In the next step, the mean of each centre with the nodes is calculated and these new means then become new centres. These same steps are repeated until the criterion function $\left(C_{r}\right)$ becomes minimum. We can find this function by the formula:

(14) $$C_{r}=\sum_{i=1}^{I}\sum_{d_{i}}\left|x_{d_{i}}-x_{i}\right|.$$

Here $\left(x_{d_{i}}-x_{i}\right)$ gives the average distance of nodes with point $x_{i}$ in cluster *i*. In the end, the node which is closest to the respective centre will then become a CH and all other nodes will become member nodes of the cluster. Performance of K mean clustering mostly depends on the ideal K value. An elbow [20] algorithm is used to identify the optimum value of K.

The above discussed scheme can be modified by incorporating the GW nodes. The chances of GW nodes in this clustering are very rare and the new scheme is called the clustering-K mean-GW. These schemes have a low maintenance cost although a central authority is needed in this case. The comparison of all these schemes are shown in Table 2. All the schemes which are discussed in Table 2 are modified by us.

### 3.2. Route Discovery

After clustering, CHs will have complete information about its clusters. In this special scenario, nodes only need to communicate with the temporary CC, so CHs can keep the routing table for CC. To find this routing table, the CH will start the route discovery process with the destination always set as a CC in which the UAV can also act as a relay. Initially, the CH will broadcast the Route Request (RREQ), and the CHs that receives this RREQ will reply with a Route Reply (RREP). The routing tables are maintained at the CHs. If a packet is transmitted successfully, then acknowledgment will be received. If this is not the case, then it will re-transmit the packet. With three consecutive packet re-transmissions, the respective CH will start the route discovery again and if the problem persists, then the CH will declare the destination inaccessible. The route discovery process is again initiated after every 250 transmissions and the same steps are repeated.

### 3.3. Routing

For routing, the node that needs to transmit will forward its packet to its respective CH. The CH will then forward this packet to the destination based on its routing table $H_{i,s_{i}}$.

In this paper, we apply RL to update the entries of the routing table to improve EE. Similar to [51], we use a linear functional approximation for the cost function. The proposed cost function is defined as:

(15) $$Cost=\beta \left(\alpha \left(\frac{d_{mn}}{max(d_{mn})}\right)+\left(1-\alpha \right)\left(\frac{d_{n(\mathrm{UAV}/\mathrm{CC})}}{max(d_{n(\mathrm{UAV}/\mathrm{CC})})}\right)\right)+\left(1-\beta \right)\left(\frac{E_{mn}}{max(E_{mn})}\right)\;$$

where, α is the weighting factor for shortest distance to the UAV and the next hop and β is the weighting factor to provide a balance between the distance and energy, and *m* and *n* are sender and receiver respectively. $d_{mn}$ is the distance between *m* and *n* while $max(d_{mn})$ is the maximum distance between *m* and *n*. Similarly $E_{mn}$ is the transmission energy between *m* and *n* while $max(E_{mn})$ is the maximum energy cost for the next hop. The above cost function consists of two sections, the distance and energy. This cost is only checked at a CH. The distance is balanced by α which considers the weight of the next hop and the corresponding distance of the next hop to UAV/CC. Choosing a minimum distance path to the UAV/CC can cause significant load balancing issues. The energy consumption of forwarding CHs in such a path will also increase significantly. On the contrary, only choosing the minimum distance path for the next hop may not always be feasible as it can increase the number of hops or the distance to the UAV. The value of α finds the tradeoff in terms of distance which is subsequently used by the parameter β to manage the tradeoff among immediate energy expenditure in the next hop and distance. For example, if a CH has connectivity to multiple CHs through which it can forward its data. The scaling factor β is proportionate to the immediate energy cost of the next hop and distance cost of different routes. The terms $\max(E_{mn})$, $\max(d_{mn})$ and $\max(d_{nUAV/CC})$ normalize the energy and distance of all the possible routing paths in the next hop.

Once the costs are calculated in the current epoch, the routing is carried out. All the steps will remain the same except after the cost function RL is applied by using the equation given below:

(16) $$RL_{mn}=\left(1-\gamma \right)RL_{mn}^{{}^{\prime }}+\gamma (Rw_{mn}+V\left(max\left(RL_{m^{{}^{\prime }}n^{{}^{\prime }}}\right)\right).$$

Here $RL_{mn}^{{}^{\prime }}$ is the previous value of $RL_{mn}$, $Rw_{mn}$ is the reward obtained from communication which in this case is 0 for an unsuccessful transmission and 1 for a successful transmission, γ is the discount factor varying between 0≤γ≤1 which tells us how much importance we want to give to the current rewards, and *V* is the learning rate varying between 0≤V≤1 that tells us to what extent these RL values are updated after each iteration. $max(RL_{m^{{}^{\prime }}n^{{}^{\prime }}})$ is the maximum calculated cost for the next hop. $\left(1-\gamma \right)RL_{mn}^{{}^{\prime }}$ takes a weight of the old $RL_{mn}^{{}^{\prime }}$ value and then by adding the learned value which is the combination of $Rw_{mn}$ and current $max(RL_{m^{{}^{\prime }}n^{{}^{\prime }}})$. This means an action is taken after looking at the old, current, and future rewards as shown in Algorithm 1.
**Algorithm 1:** Reinforcement Learning Algorithm.
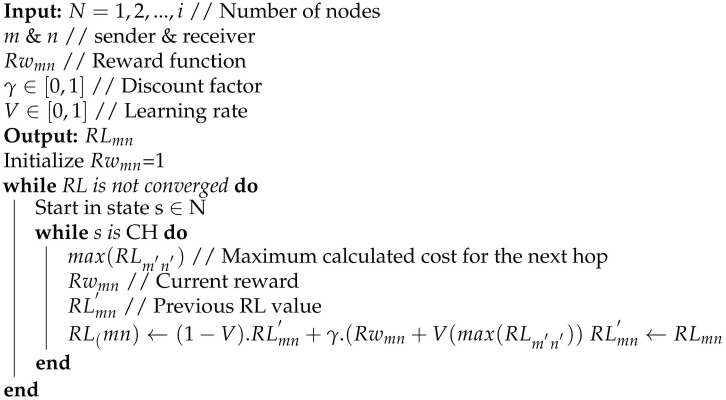


### 3.4. Control Overhead

This subsection summarizes the cost of control overhead in clustering and non-clustering schemes. The control overhead in the above schemes can be categorized as a beacon message overhead, clustering overhead, and routing overhead.

- **Beacon messages overhead**: These beacon messages are sent by the nodes to find the information about their respective neighbors. The number of beacon messages sent are dependent on the number of nodes, N, in the environment. The nodes which are in its vicinity will reply. These beacon messages are resent after every 10 s to renew the neighbor’s information. Since there is no mobility in our scenario the neighbor change is only possible due to DNs.
- **Clustering overhead**: To calculate the clustering overhead, we categorize the clustering schemes as centralized and decentralized schemes. The schemes that employ K mean clustering are centralized schemes because they require the location information of all the nodes. We assume that after the exchange of beacon messages, location information of all the nodes is forwarded/relayed to the CC. The CC performs K mean clustering and inform the nodes about their clusters and CHs. Here, we do not consider the overhead of passing location information to CC and K mean information back to the nodes from CC. This approximate overhead can be readily found from the achievable capacity [52]. The schemes that employ clustering energy are distributed schemes. The clustering overhead is calculated when a node broadcasts control packets to declare itself as the CH. The receiving nodes will reply accordingly as discussed in Section 3.1.1. These control packets depend on the number of clusters *i* and the number of CMs in each cluster.
- **Routing overhead**: Once the CHs are formed, this step includes the amount of control overhead involved in the discovery of neighboring CHs and the routing path. It is assumed that the routing tables are only maintained at the CHs, which will reduce the overall routing overhead. For route discovery, the CHs will send the control packets to the neighboring CHs which will then forward the control packets all the way to the CC. The CC will confirm the routing path for each CH through a reverse response as discussed in Section 3.2.

## 4. Performance Analysis

**Figure 4:**
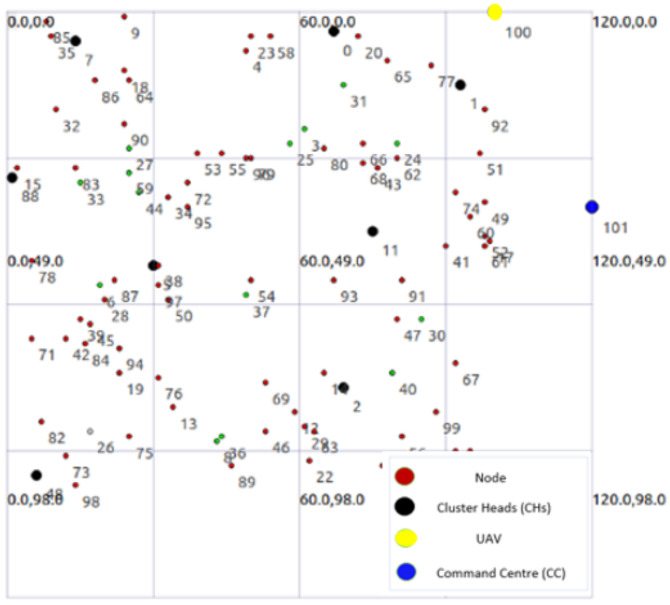
Topology of the network in NS-3.

For simulations, we consider an area of 100×100 m${}^{2}$ with N=100 nodes is considered as shown in Figure 4 with the UAV placed at the edge and CC placed further away for safety reasons. The UAV and CC are static and their placement allows a limited number of nodes in their transmission range. These nodes (GW and CH) are consequently used to reach the UAV and CC in a multi-hop manner resulting in rapid depletion of their battery.

In Figure 4, the blue color is used for CC, the yellow color is used for UAV, the black color is used for CHs, the green color is used for GWs, the red is used for CMs, and the white color is used for Dead Nodes (DNs). The white nodes are not active and unable to communicate due to low or no $E_{initial}$. This topology is obtained after running NS-3-based simulations for several rounds. The simulation parameters are shown in Table 3.

**Figure 5:**
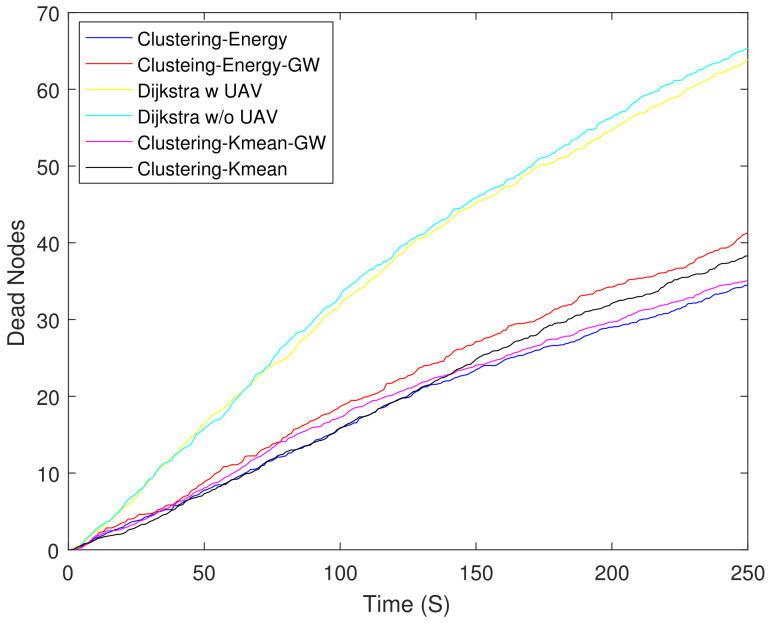
Number of dead nodes.

For a fair comparison, we include the results of non-clustering schemes based on Dijkstra [53] along with schemes discussed in Section 3.1. To get a better insight into the schemes performance, we assigned random energy varying between 0 and 1 J to all the nodes. Figure 5 shows the number of DNs where the total number of DNs is very high because nodes have random $E_{initial}$ and they die quickly. The figure shows that both Dijkstra-based schemes had the highest number of DNs. This is approximately 57% higher compared to clustering-energy at 250 s, respectively.

**Figure 6:**
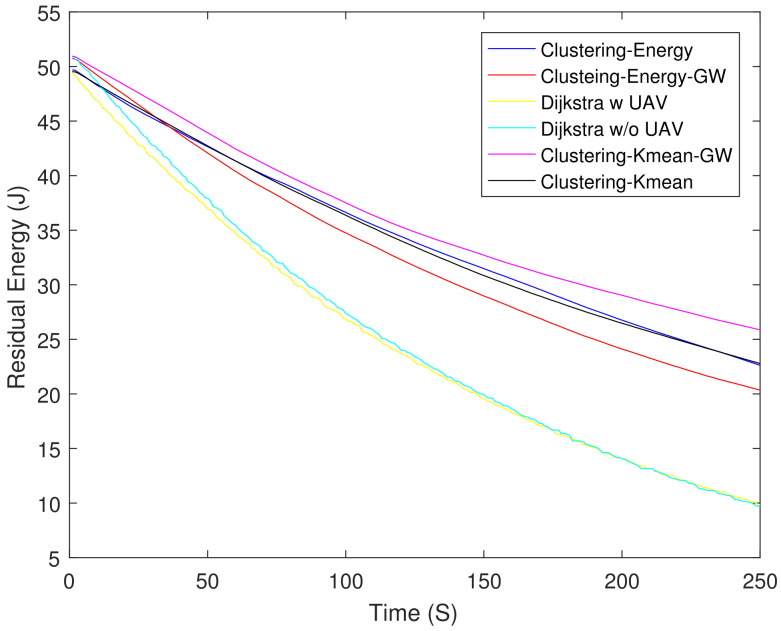
Residual energy.

**Figure 7:**
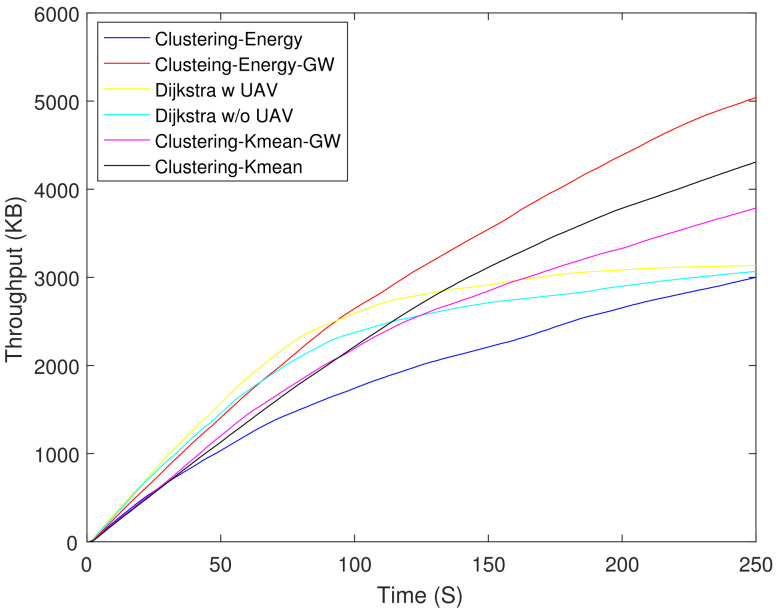
Throughput.

**Figure 8:**
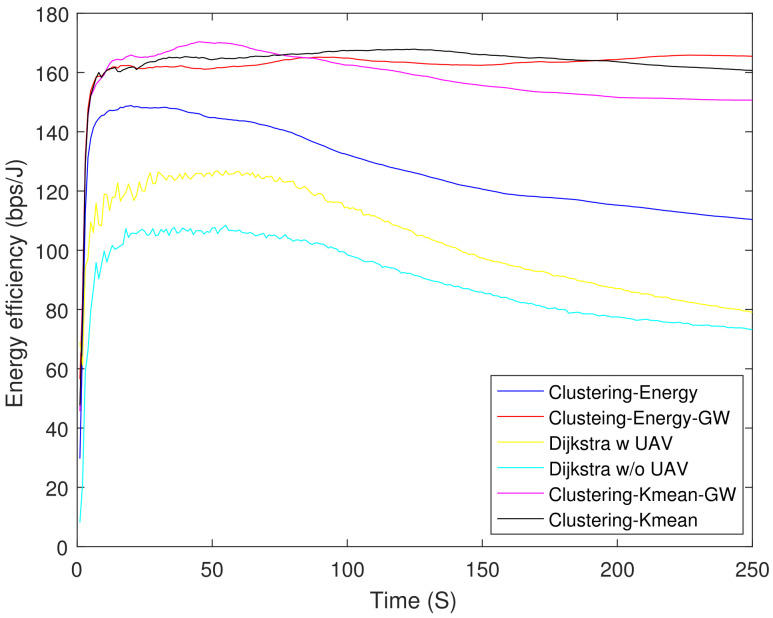
Energy efficiency.

**Figure 9:**
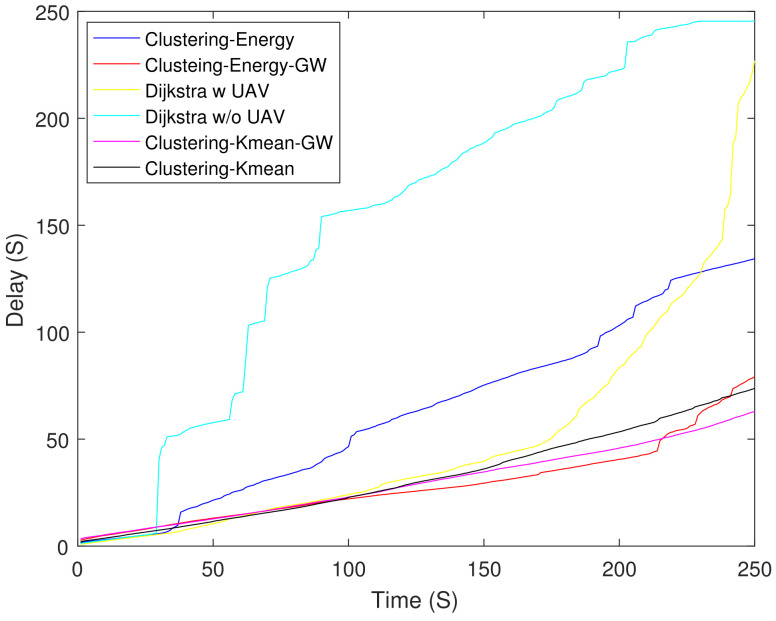
End-to-end delay.

Figure 6 shows the residual energy of different schemes. As expected, the Dijkstra-based scheme had the lowest residual energy at 250 s. The Dijkstra-based scheme consumed 61% higher energy as compared with clustering-K mean-GW at 250 s. Figure 7 shows the throughput of all the schemes. Until 90 s, Dijkstra with UAV shows the highest throughput, but after 90 s its curve starts to saturate due to the increasing number of DNs. Clustering-energy-GW has the highest throughput after 250 s i.e., 38 and 40% higher as compared to Dijkstra with UAV and clustering-energy, respectively. Figure 8 shows the EE of different schemes. The clustering-K mean-GW has highest throughput per unit of energy until 35 s. Beyond 35 s, clustering-K mean shows highest throughput starting from 75 s to 180 s. Both the Dijkstra-based schemes have the lowest EE and decrease further with time. The clustering-K mean has a 29 and 45% higher EE as compared to clustering-energy and Dijkstra without UAV. Figure 9 shows end-to-end delay. The delay is very high in all the schemes because of the high hop count. In comparison, Dijkstra without UAV shows the highest delay followed by Dijkstra with UAV. Clustering-K mean-GW offers a 75% lower delay when compared with Dijkstra without the UAV.

**Figure 10:**
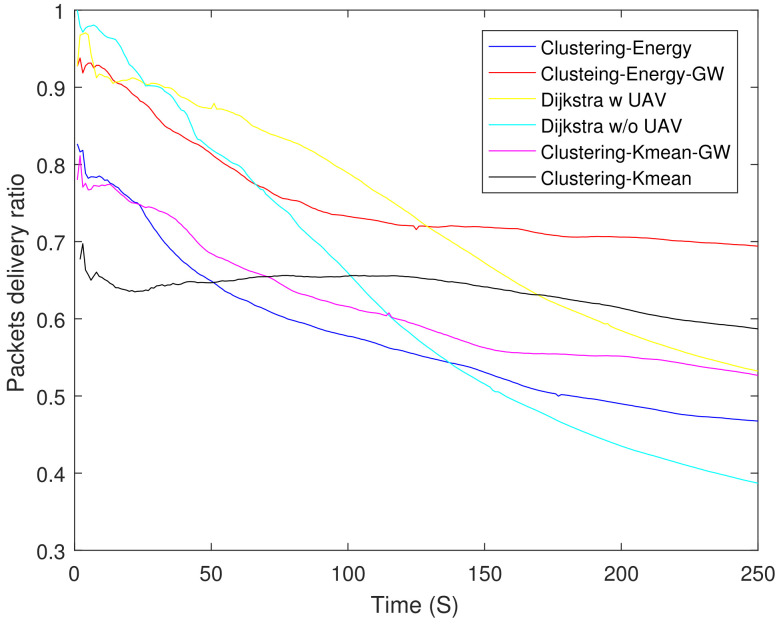
Packet delivery ratio.

Figure 10 shows Packet Delivery Ratio (PDR) of different schemes. In the beginning, both the non-clustering schemes (Dijkstra) have a higher PDR upto 75 s. Afterwards their performance degrades and Dijkstra w/o UAV has the lowest PDR. Compared to (Dijkstra), the PDR of clustering schemes decays slowly. The clustering-energy-GW have the highest PDR after 150 s and it is 9.2% higher than that of clustering-K mean at 250 s.

### 4.1. Reinforcement-Based Routing

**Figure 11:**
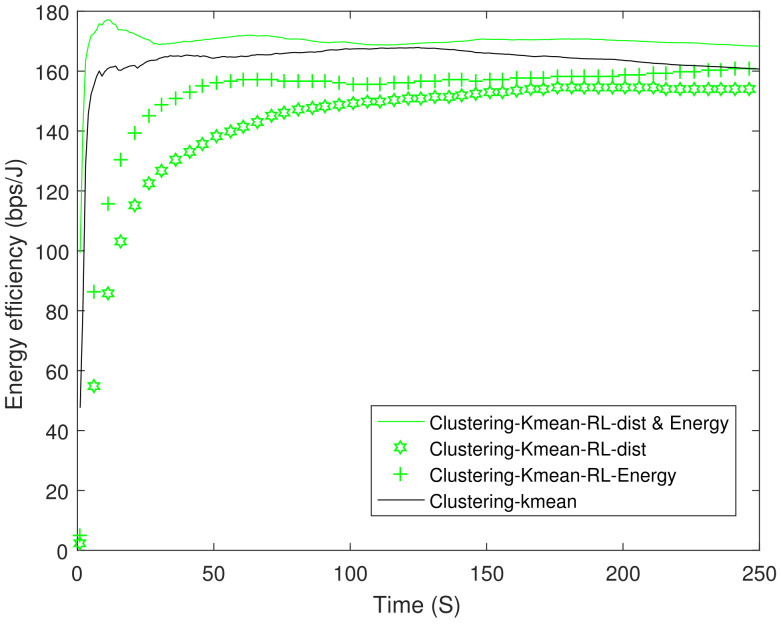
Energy efficiency of the reinforcement learning scheme. RL: Reinforcement Learning.

**Figure 12:**
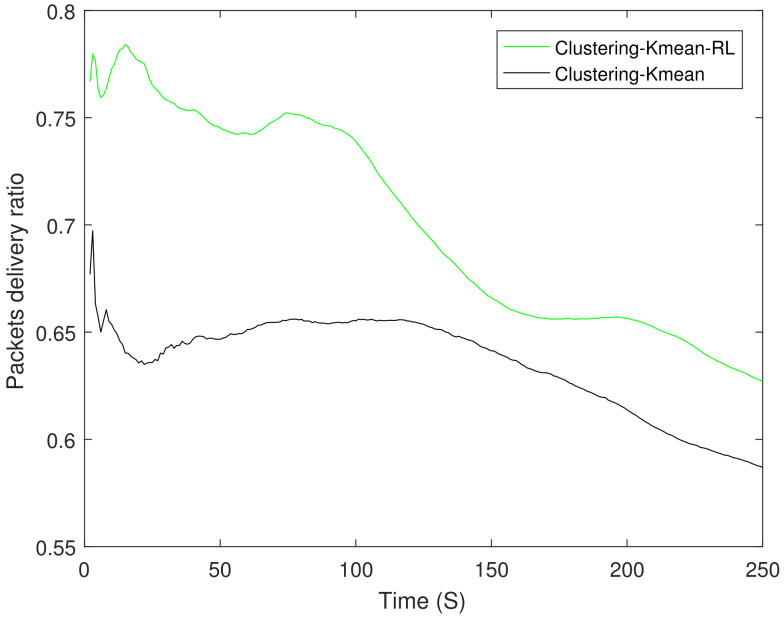
Packet delivery ratio of the reinforcement learning scheme.

From earlier results we observed that clustering-K mean performs the best in EE, therefore, we apply RL on the best performing scheme. Figure 11 shows the EE between the best performing scheme clustering-K mean and clustering-K mean-RL. The results show an improvement of up to 15% at 10 s when compared with the underlying scheme. In addition, we have also compared RL-based distance and energy only variant of Equation (15). Its worth mentioning that RL-based distance only variant performed much better compared to both conventional Dijktsra and Dijkstra with UAV. Figure 12 shows PDR between the clustering-K mean and its RL variant. RL-based K mean variant shows at least a 4% improvement.

**Figure 13:**
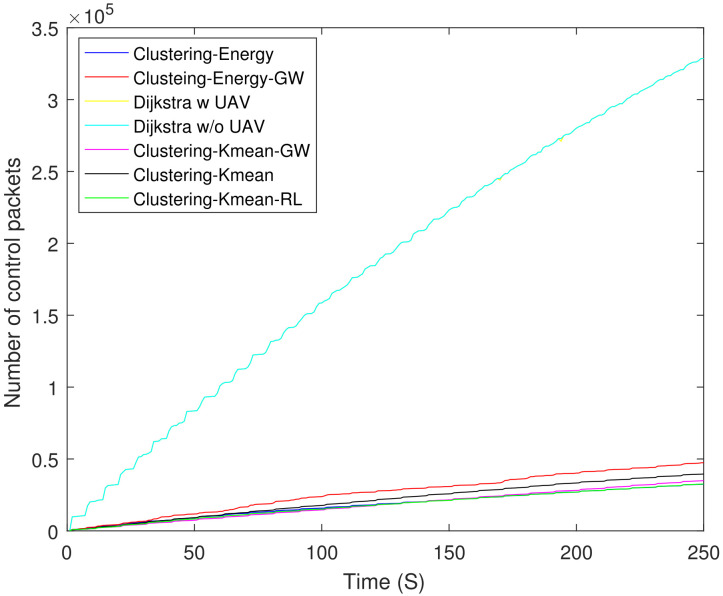
Control packets sent by all the schemes.

**Figure 14:**
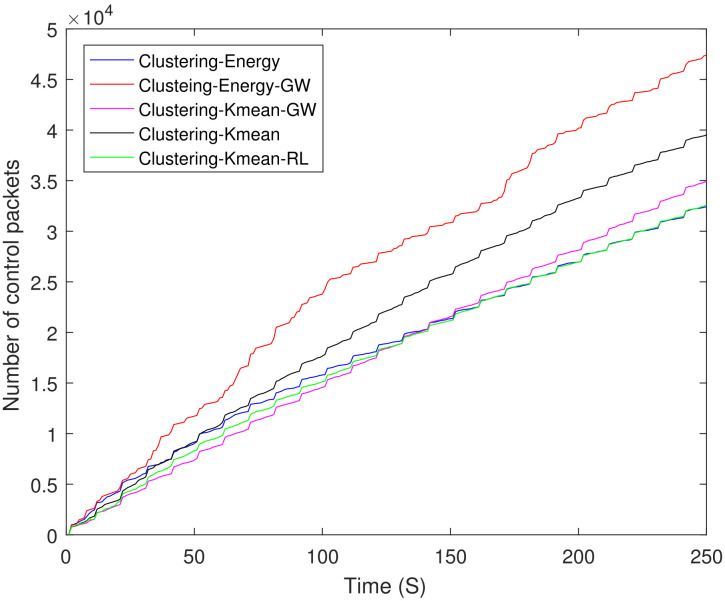
Control packets sent by different clustering schemes.

Figure 13 shows a comparison of the total number of control packets sent by all the schemes including clustering and Dijkstra-based schemes. Both Dijkstra-based schemes transmit almost the same number of control packets. It is interesting to observe that the control overhead of Dijkstra-based schemes is approximately 90% higher than the clustering-energy-GW. Figure 14 shows the same comparison between different clustering schemes. The control overhead of clustering-energy-GW is 32% higher when compared to clustering-energy, clustering-K mean-GW, and clustering-K mean-RL.

**Figure 15:**
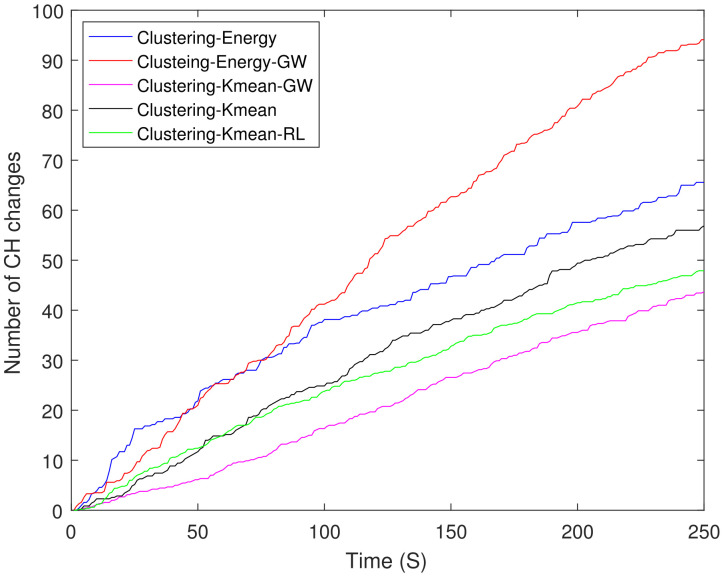
Number of CH changes in different clustering schemes.

Figure 15 shows the number of CH changes in different clustering schemes. The energy-based schemes show the highest number of CH changes when compared with the K mean clustering schemes because of their distributed nature. Clustering-energy-GW shows 53% more CH changes when compared with clustering-K mean-GW.

### 4.2. Combined RL and UAV(s) Trajectory Optimization

**Figure 16:**
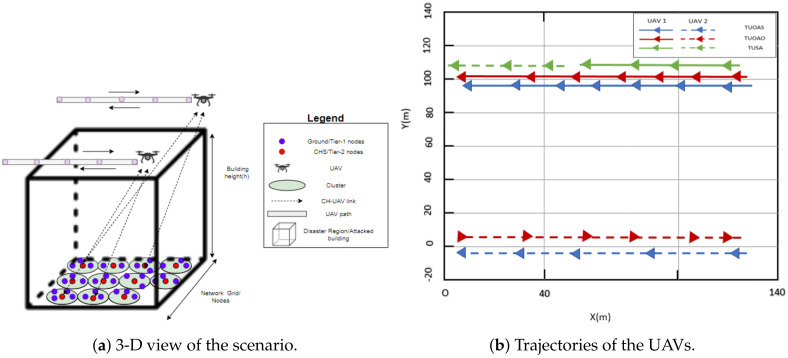
Trajectories of UAVs around the disaster area.

In the previous sections, the UAV was considered to be a static node. However, the UAVs act as flying relays and with an adequately designed flight trajectory they can provide uniform coverage to all the CHs, thus increasing EE. In this paper we consider the placement of single and multiple UAVs and analyze the impact of their trajectory. The purpose of multiple UAVs is to decrease the number of hops and provide an improved EE. In the disaster scenarios, the energy of the devices is very critical so to further improve EE and connectivity, a multiple-UAV scenario is applied. The speed of UAVs in motion is 2.70 m/s. The transmission range of a UAV is 60 m. If the CHs falls in the transmission range of two UAVs, the CH will choose the UAV closest to the CC. The RL is used to find the best routing paths in the scenario given in Figure 16. Figure 16a provides three dimensional view of the disaster scenario and Figure 16b shows the trajectory paths of UAV’s around the disaster scenario. We consider pre-defined UAV flight paths around the area which are discussed below.

- **Two UAVs on Opposite Axis and Same direction (TUOAS)**: In this scheme, the two UAVs are placed at the edge of the building. Both the UAVs are placed on the opposite corners of the building. They start moving from the same side of the building, as shown in Figure 16. Both these UAVs are moving in parallel to each other but the direction they are following is the same. The trajectory they are following is along the straight line alongside the building. When they reach the opposite corner of the building, they will follow the same path backwards. The CHs that are in the range of any of these UAVs will send there packets through the respective UAV. Thus, the Equation (15) for the two UAVs will be modified as:
  (17) $$Cost_{1}=\beta \left(\alpha \left(\frac{d_{m_{1}n_{1}}}{max(d_{m_{1}n_{1}})}\right)+\left(1-\alpha \right)\left(\frac{d_{n_{1}\left({\mathrm{UAV}}_{1}\right)}}{max(d_{n_{1}\left({\mathrm{UAV}}_{1}\right)})}\right)\right)+\left(1-\beta \right)\left(\frac{E_{m_{1}n_{1}}}{max(E_{m_{1}n_{1}})}\right).\;$$
  Here $Cost_{1}$ is the cost associated with UAV${}_{1}$ in which $m_{1}$ and $n_{1}$ are the sender and receiver nodes associated to the UAV${}_{1}$:
  (18) $$Cost_{2}=\beta \left(\alpha \left(\frac{d_{m_{2}n_{2}}}{max(d_{m_{2}n_{2}})}\right)+\left(1-\alpha \right)\left(\frac{d_{n_{2}\left({\mathrm{UAV}}_{2}\right)}}{max(d_{n_{2}\left({\mathrm{UAV}}_{2}\right)})}\right)\right)+\left(1-\beta \right)\left(\frac{E_{m_{2}n_{2}}}{max(E_{m_{2}n_{2}})}\right).\;$$
  Similarly $Cost_{2}$ is the associated cost with the UAV${}_{2}$. While $m_{2}$ and $n_{2}$ are the sender and receiver nodes associated to the UAV${}_{2}$. In the case, sender *m* is in the direct range of CC, the cost will be calculated using Equation (15). The above equations can also be used for all other two UAV schemes presented below.
- **Two UAVs on Opposite Axis and Opposite Direction (TUOAO)**: In this scheme two UAVs are placed at the opposite corner of the building as shown in Figure 16. Both UAVs moves along a straightline alongside the building towards their respective direction. By moving in this way they will help in maximize the coverage area of the building affected by the disaster. When a UAV reaches the edge of the building it will follow the same path backwards and it keeps on doing this till the end of the simulation.
- **Two UAVs moving on Same Axis (TUSA)**: In this scheme, both the UAVs were placed on the same axis separated by 60 m, as shown in Figure 16. Both the UAVs move in the same direction and on reaching there respective endpoint they follow the same path backwards. The maximum separation between them remains the same.
- **Single UAV in motion (SU)**: In this scheme, we placed a single UAV at the corner of the building. The UAV moves alongside the building and traverses the same path on its way back from the end of the building.

**Figure 17:**
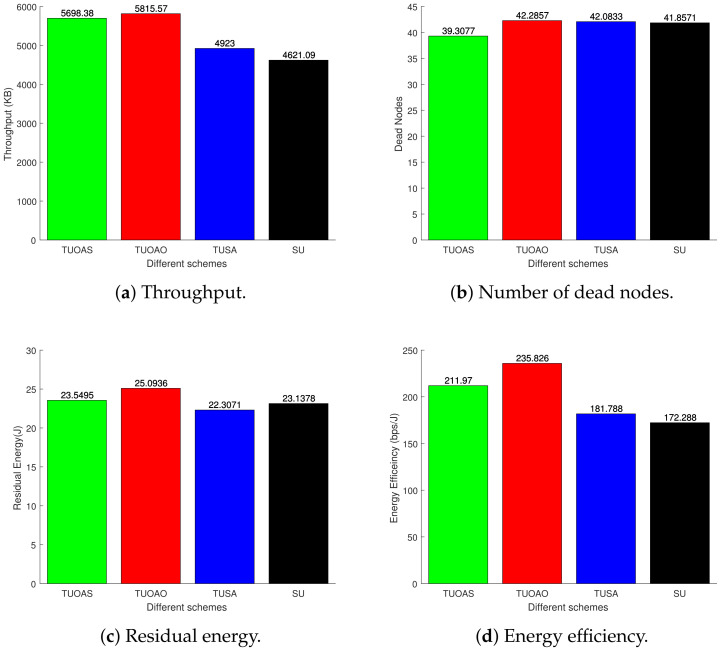
Results after 250 s.

**Figure 18:**
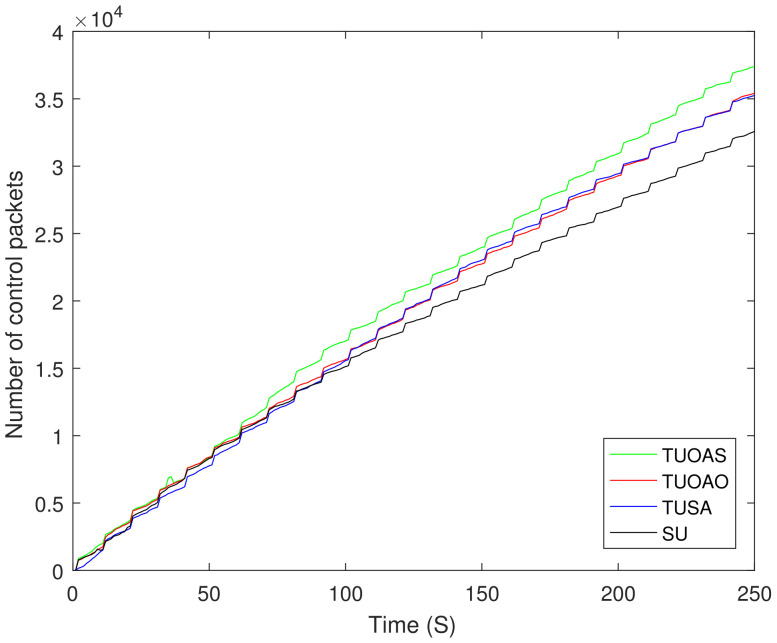
Number of control packets.

**Figure 19:**
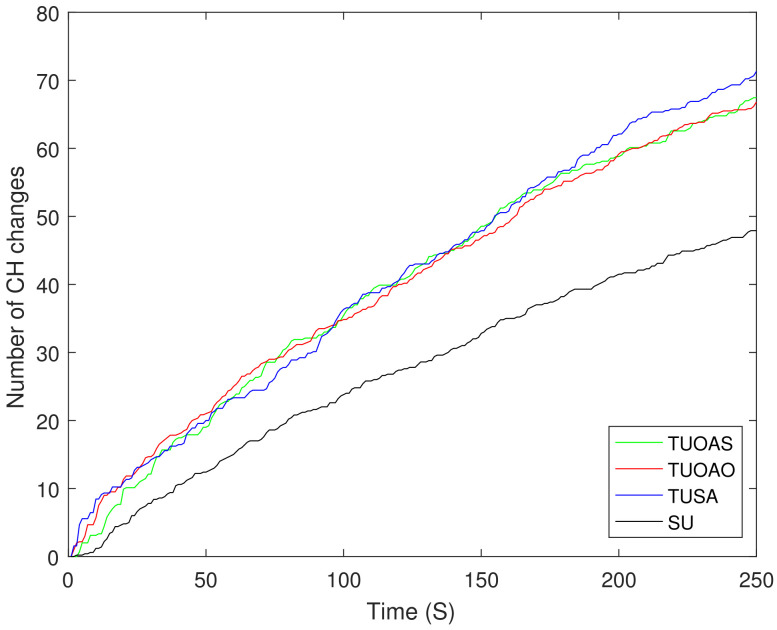
Number of CH changes.

We have considered variable energy for the nodes varying between 0 and 1 J. The bars in Figure 17a show that all the schemes achieve their maximum throughput after 250 s. The TUOAO records the highest throughput and provide 2%, 15%, and 20% gain when compared with TUOAS, TUSA, and SU, respectively. Figure 17b presents the number of DNs for each scheme. The difference between the schemes TUOAO, TUSA, and SU was minor and only TUOAS showed 7% lesser DNs compared to the other three schemes. Figure 17c shows the residual energy after 250 s. TUOAO has the most residual energy whereas TUOAS was second with 6% lower residual energy, and SU and TUSA are third and fourth, respectively. Figure 17d shows EE. All the schemes present a higher performance compared to the SU case. TUOAO presents the highest gain in EE, whereas the EE of TUOAS is 10% lower. The EE of TUSA and SU is 23% and 27% lower when compared to TUOAO. Figure 18 compares the number of control packets sent for different trajectory schemes. The scheme with a single UAV sends the least amount of control packets, 14% lower than that of TUOAS. Figure 19 shows the number of CH changes against time. The schemes with two UAVs have a higher number of CH changes. This is mainly due to the routing overhead induced by the movement of the UAVs, which result from frequent changes in routing paths. Intuitively, the SU scheme in comparison has 50% fewer CH changes than the TUOAS.

## 5. Conclusions

In this work, multiple routing schemes were evaluated for a man-made disaster scenario. The main concern in these scenarios is the network lifetime achieved through EE. As expected, the simulation results showed clustering schemes had a longer network lifetime when compared with non-clustering schemes. In terms of throughput, clustering-energy-GW had 40% higher throughput than clustering-energy. However, in terms of EE, clustering-K mean showed the best performance. We then applied RL on the clustering-K mean which further improved the EE by 15% at 10 s. Inclusion of multiple UAVs further improved EE, however, the amount of improvement was highly dependent on the trajectory of these UAVs. In a rectangular disaster area, maximum EE was achieved when the UAVs started scanning linearly from the opposite ends of the building while maximizing the coverage. However, this came at the cost of increased routing overhead and cluster changes. In future, we plan to get a holistic picture by incorporating the impact of control packets. It is also possible to explore the use of localization techniques to find the exact location of the nodes, which is useful in planning multi-UAV deployment.

## Figures and Tables

**Table 1:** Table of symbols.

Name	Symbol	Name	Symbol
Number of nodes	*N*	Number of clusters	*i*
Cluster radius	$R_{max}$	CH Tx radius	$T_{max}$
K value	*K*	Average distance of each K	$W_{k}$
Distance	*d*	Transmissions	*T*
Reward function	Rw	Learning rate	*V*
Discount factor	γ	Path loss	PL
Carrier frequency	$f_{c}$	Number of walls	$n_{w}$
Number of floors	$n_{f}$	Floor loss	$F_{l}$
Throughput	*r*	Path	ρ
Routing matrix	*H*	Packet size	*L*
Energy Efficiency	EE	Delay	δ
Transmitting power	$P_{t}$	Bandwidth	*B*
Noise power	$N_{o}$	Packet size	*L*
Beta	β	Alpha	α

**Table 2:** Comparison between clustering schemes. GW: Gateway.

Scheme Name	Clustering Type	Clustering Overlapping	Gate Way	Location Awareness
Clustering-energy	Energy based	High	No	Not required
Clustering-energy-GW	Energy based	High	Yes	Not required
Clustering-k mean	K mean	Low	No	Required
Clustering-k mean-GW	K mean	Low	Yes	Required

**Table 3:** Simulation parameters.

Parameter	Values
Number of Devices (N)	100
Network Grid	100 m × 100 m
CC Placement	(120,35) m
UAV Placement (Initial)	(100,0,10) m
Size of Data Packet (L)	1024 bytes
Header Size	40 bytes
Initial Power Level	0 to 1 J
$E_{Tx}$	50 nJ/bit
$E_{Rx}$	50 nJ/bit
Threshold	4.35 mJ
Cluster Range, $R_{max}$	30 m
CH Tx Range, $T_{max}$	45 m
Distance b/w UAV and CC	60 m
Max Transmissions in a Round ($N_{Tx}$)	5
$I_{max}$	1024
α	0.5
β	0.5
Discount Factor (γ)	0.8
Learning Rate (*V*)	0.4

## Data Availability

Not applicable.
